# Prolonged Visual Experience in Adulthood Modulates Holistic Face Perception

**DOI:** 10.1371/journal.pone.0002317

**Published:** 2008-05-28

**Authors:** Adélaïde de Heering, Bruno Rossion

**Affiliations:** Unité Cognition et Développement et Laboratoire de Neurophysiologie, Université Catholique de Louvain, Louvain-la-Neuve, Ottignies, Belgium; University of Maryland, United States of America

## Abstract

**Background:**

Using the well-known composite illusion as a marker of the holistic perception of faces, we tested how prolonged visual experience with a specific population of faces (4- to 6-year-old children) modulates the face perception system in adulthood.

**Methodology/Principal Findings:**

We report a face composite effect that is larger for adult than children faces in a group of adults without experience with children faces (“children-face novices”), while it is of equal magnitude for adults and children faces in a population of preschool teachers (“children-face experts”). When considering preschool teachers only, we observed a significant correlation between the number of years of experience with children faces and the differential face composite effect between children and adults faces. Participants with at least 10 years of qualitative experience with children faces had a larger composite face effect for children than adult faces.

**Conclusions/Significance:**

Overall, these observations indicate that even in adulthood face processes can be reshaped qualitatively, presumably to facilitate efficient processing of the differential morphological features of the frequently encountered population of faces.

## Introduction

Faces are thought to be processed by neural mechanisms in human adults that are finely tuned to the morphological characteristics of the population of faces present in the visual environment. More specifically, own-race faces are better recognized than other-race faces, a phenomenon called the “other-race effect” in the face processing literature (ORE, 1; for a recent meta-analysis see 2). In the same vein, it has also been shown that same-age faces are better recognized than other-age faces (the “other-age effect”, OAE, 3–5).

The ORE is related to visual experience. It appears early in development (three months, 6; between six and nine months, 7), but it can be reversed if the perceptual system is exposed to other-race faces rather than same race faces during childhood [8]. However, it remains unclear whether the ORE can be reversed or significantly attenuated in adults following natural visual experience or expertise training with other-race faces [2]. Regarding the processing of other-age faces, a recent study demonstrated that preschool teachers with extensive visual experience with children faces did not present the OAE, in contrast to adults who had little experience with children [5]. These observations suggest that experience acquired in adulthood with other-age faces can modulate the OAE.

Rather than concentrating on quantitative differences (i.e. recognition performance measures), recent studies have investigated the nature of the differential processes for experienced and non-experienced faces. Using tasks measuring the interdependence of facial features such as the whole-part superiority effect [9] and the face composite effect [10; see below], it has been shown that adult observers are processing other-race faces less holistically than same-race faces [11]–[13]. This qualitative difference may be related to the ORE. However, since the adult participants in these studies had been more or almost exclusively exposed to same-race faces during their entire life, it remains unknown whether visual experience with other-race faces can reshape face processes qualitatively, in particular in adulthood. That is, whether extensive experience with other-race faces would lead to an increase in holistic processes for new unfamiliar faces of this category.

**Figure 1:**
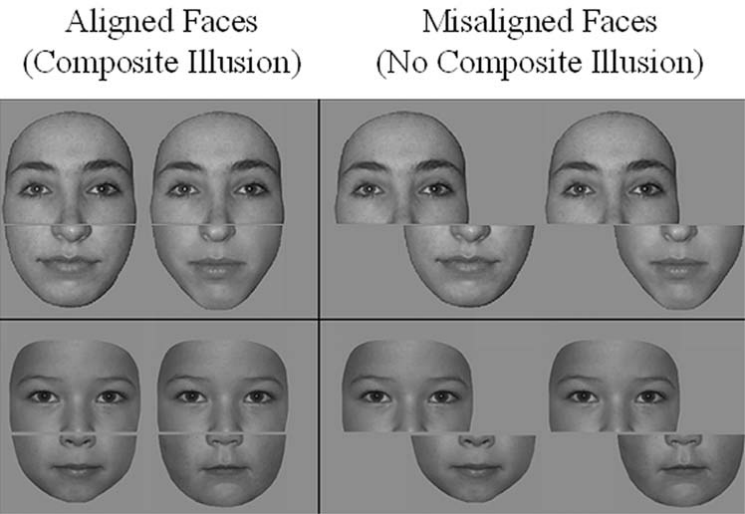
Left: The face composite illusion on adult and children faces. The top parts (above the gap) of the faces are identical but perceived as slightly different due to the integration with the distinct bottom parts. Right: When the top parts and bottom parts are misaligned, the illusion vanishes. Faces were presented sequentially.

The present study addressed this question of the plasticity of face processes in adulthood by contrasting the processing of same- and other-age faces in experts (preschool teachers) and novices. To test the hypothesis of a qualitative modulation of face processes following visual experience in adulthood, we measured the face composite effect [10] as a marker of the integration of facial features, i.e. holistic face perception. The face composite effect as applied to unfamiliar faces refers to the perception of differences in two identical top parts (i.e. all information located above the nose) if they are aligned with different bottom parts (e.g. [14]–[16]; Figure 1). This visual illusion depends on early visual experience [15], is present as early as 4 years of age [17], and is subtended by right hemisphere occipito-temporal areas responding preferentially to faces [18]. Here, we tested the strength of this illusion in an individual matching task of unfamiliar adult and children faces, both in preschool teachers (experts) and in age-matched participants having little experience with children faces (novices). In line with studies on the ORE, we hypothesized that the face composite illusion would be stronger for adult faces than children faces in novices, but of equal magnitude for the two sets of faces in experts with children faces. The results support this hypothesis, but also demonstrate a correlation between the amount of visual experience with children faces and the differential face composite effect for children and adult faces in experts.

## Results

We performed distinct analyses of variance (ANOVAs) on participants' response accuracy (percentage of correct responses on same trials) and correct response times (ms) with the *alignment* of the face parts (aligned *vs.* misaligned) and the *type* of face (adult *vs.* children faces) as a within-subjects factor, and the *group* (novices *vs.* experts) as between subjects factor.

For trials of interest (“same” decision, see 16), there was a main effect of the *alignment* of the face parts on accuracy (F_1,34_ = 69, p<.001), participants being better in the misaligned (MS) as compared to the aligned (AS) condition. The three-way interaction on correct responses between the *group*, the *type* of face and the *alignment* of the face parts failed to reach significance (p>.2). There was also a composite face effect on correct response times since there was a main effect of the *alignment* of the face parts (F_1,34_ = 97.9, p<.001). For response times, the three-way interaction between the *group*, the *type* of face and the *alignment* of the face was significant (F_1,34_ = 6.14, p<.02). Post-hoc t-tests contrasting the magnitude of the normalized composite effect ((MS−AS)/(MS+AS)) for adult and children faces revealed a much larger composite effect for adult as compared to children faces in novices (t(17) = −3.643, p = .002), but no significant difference in experts (t(17) = .001, p = 1) (see Table 1).

**Table 1:** Mean response times and standard errors are shown for matching “same” top halves of adult and children faces, with the bottom part aligned (AS) or misaligned (MS), in experts and novices.

Groups	N	Adult faces			Children faces		
		Aligned (AS)	Misaligned (MS)	Composite effect (MS−AS/MS+AS)	Aligned (AS)	Misaligned (MS)	Composite effect (MS−AS/MS+AS)
Experts	18	732±28	648±24	−0.059±0.01	719±27	635±19	−0.060±0.01
Novices	18	742±40	621±32	−0.088±0.01	697±42	630±40	−0.051±0.01

**Figure 2:**
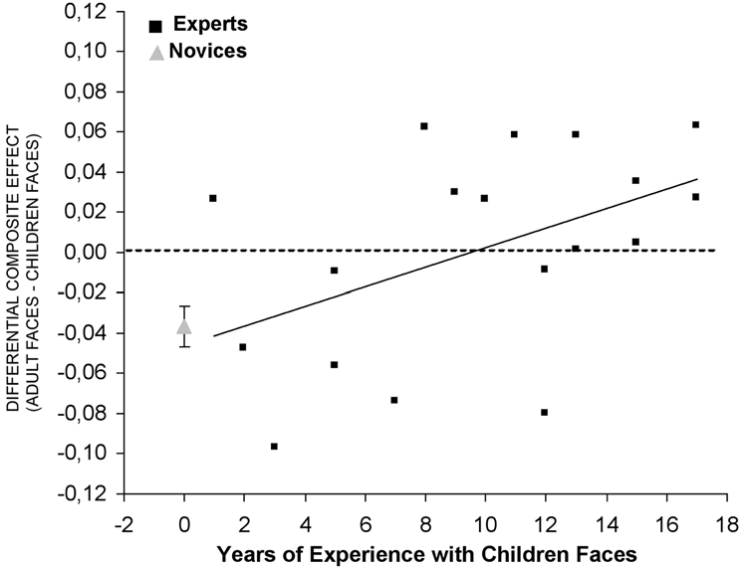
Participants' differential composite face effect (composite face effect on adult faces–composite face effect on children faces) plotted as a function of the amount of experience with children faces.

Interestingly, the magnitude of the differential face composite illusion calculated as the difference between the composite ratios for adult and children faces was significantly correlated (r = .47, p<.05) with the number of years of visual experience with children faces. More precisely, the differential composite effect becomes larger for children as compared to adult faces after about 10 years of experience with children faces (see Figure 2).

## Discussion

In summary, we found that both experts and novices are better and faster at matching two top parts of faces when they are misaligned with their respective bottom parts than when they are aligned with these bottom parts. This was found for adult faces and, for the first time to our knowledge, for children faces tested with adult participants. More interestingly, when considering differential response times between aligned and misaligned conditions as in previous studies [e.g. 10], [12], [15], [19], there was a significantly stronger composite face illusion for adult compared to children faces in novice participants, while it was of equal magnitude in experts with children faces. This indicates a stronger holistic perception for adult faces in novices, but an equal amount of holistic face perception for adult and children faces in experts. Finally, the correlation analysis suggests that the magnitude of the experts' composite face illusion for children faces is directly related to the amount of experience with this population of faces.

The observation of a face composite effect for children faces even in novice observers is entirely consistent with previous work, in which holistic face processing is significant for other- race faces [11]–[13]. It indicates that the holistic face processes are coarsely defined [19], since they can be applied to faces that have a different morphology than the population of faces frequently encountered in the visual environment [20]. However, response times data collected in novices suggest that these processes are also finely tuned by experience: holistic processing is smaller for faces with which we have less visual experience, namely children faces here. This finding is also consistent with the observation of smaller inversion effects–an indirect measure of holistic processing [e.g. 21]–for other-race than same-race faces [22]–[23]; [but see 24], as well as for children as compared to adult faces in novice observers [5].

Here, most interestingly, we found that the greater amount of holistic processing for adult than children faces was no longer present in the population of participants with extensive experience at processing children faces, and was even reversed in favor of children faces following 8–10 years of extensive experience with these faces (see Figure 2). These observations indicate that even when the face processing system is fully matured [e.g. 25] and fine-tuned to process adult faces, it can be modulated by visual experience to accommodate a population of faces with different morphological features (children faces, 20). These findings also indicate that face representations are most likely to be organized around a single “prototypical” representation of faces [e.g. 26] since teachers' face composite effect was larger for children faces after about 10 years of experience, rather than being equal for both of these face categories.

An equal performance at recognizing children faces than adult faces in preschool teachers as reported previously [5] could potentially be due to multiple factors (better perceptual sensitivity, more robust mnesic representations, attentional/emotional factors …). In contrast, the findings reported here indicate a qualitative difference (holistic face processing) that is measured implicitly (i.e. participants are told to focus on one half of the face and their judgment is influenced by the other half). Moreover, the face composite effect reflects a visual illusion (see Figure 1) that has a perceptual locus [18]–[19]. Hence, these observations suggest that extensive natural experience with children faces modified perceptual face processes. In addition, all the faces used were unfamiliar to the participants, such that a potential role of emotional factors in the differential effect reported is unlikely.

One potential limitation of the present study is that visual experience of the participants with children faces was not assessed in an independent task prior to testing. However, as done previously (Kuefner et al., in press), the two groups were highly contrasted, such that the teachers had a large amount of quantitative and qualitative (i.e. individual) experience with children faces, while the novices had almost no experience at all with this face category. Moreover, the two groups were matched in age and gender, and appeared to differ only in terms of their relative experience with children faces. Finally, the fact that the magnitude of the differential face composite effect was not only larger in preschool teachers but also correlated with the amount of experience accumulated with children faces reinforce the validity of our conclusions.

In summary, in line with previous evidence of visual plasticity in adulthood [27], these observations indicate that even when holistic perceptual face processes are fully matured and finely tuned to the morphology of adult faces, they can still be modulated to accommodate to a population of faces presenting different morphological features.

## Materials and Methods

### Participants

Eighteen female preschoolers' teachers (33±4 years of age, all right-handed) and 18 female participants inexperienced with children faces (31±4 years of age, all right-handed) took part to the experiment. Questionnaires indicated that preschoolers' teachers could be considered as “children-face experts” since they have had daily contacts (10 months a year, 6–8 hours during 5 days a week) with 4- to 6-year-old children for at least one full year (range: 1–17 years of teaching). Besides having accumulated quantitative experience with children faces, they were also considered as having developed qualitative experience with this face category since they have to process these faces at the individual level on a daily basis. We also ensured that “children-face novices” did not have children, knew personally only 1 to 3 children of that age, and were not exposed to children faces in their professional activities. All participants had normal or corrected-to-normal vision.

### Stimuli

All the facial stimuli were full-front views of children (4 to 6 years of age) and adults (18 to 25 years of age) posing with neutral expression, and being unfamiliar to the participants. These face stimuli were equalized for global luminance. Their external features were removed. The pictures subtended approximately 10° by 8° of visual angle in the aligned condition and were displayed on a computer screen using E-Prime 1.1.

To create the composite set of faces (see Figure 1), the original stimuli (38 children faces and 38 adult faces) were divided into top and bottom segments by dividing them in the middle of the nose using Adobe Photoshop 7.0. The segments of these original stimuli were either aligned or misaligned in reference to the initial demonstration [10] and our previous work with composite face stimuli [e.g. 12], [ 16]–[17], as well as separated by a gap of 1 pixel. Then, for each original stimulus, two composite face stimuli were constructed by joining the top segment of the original face to the bottom segment of another face stimulus of the same gender: one in which the two halves were aligned, and one in which they were misaligned. These faces were used in ‘same’ (76 AS and 76 MS) trials. Two additional composite stimuli were also constructed, one aligned and one misaligned, having both the top and the bottom part different from the original face. These were used in ‘different’ (76 AD and 76 MD) trials. All the trials were constructed by associating an original (first face) and a composite (second face) stimulus, the latter being extracted from the AS, MS, AD or MD condition.

### Procedure

Participants were tested individually, at a distance of 50 cm from a computer screen. The experiment consisted of 304 experimental trials (76 AS; 76 AD; 76 MS; 76 MD) randomly displayed in 4 blocks of 76 trials. Within a block, half of the trials were composed of children faces, the other half of adult faces. Participants were shown a fixation cross appearing in the middle of the screen and were instructed to focus on the top part of the face appearing right above the fixation cross in order to decide as quickly as possible if the 2 top parts were identical (left keyboard response key; AS and MS trials) or not (right keyboard response key; AD and MD trials). The order of the response keys was counterbalanced across participants. Before starting, each participant was familiarized with the experiment through 10 practice trials. Feedback was provided on the practice trials but not on the experimental trials. Each trial started with a fixation-cross presented in the middle of the screen for 300 ms followed by a blank screen for 200 ms and a first face for 600 ms. After a second 300 ms blank screen, a probe composite stimulus was presented until the participant's “same” or “different” judgment. The inter-trial interval was 1000 ms.
